# Positive mental health literacy: development and validation of a measure among Norwegian adolescents

**DOI:** 10.1186/s12889-017-4733-6

**Published:** 2017-09-18

**Authors:** Hanne Nissen Bjørnsen, Mary˗Elizabeth Bradley Eilertsen, Regine Ringdal, Geir Arild Espnes, Unni Karin Moksnes

**Affiliations:** 1 Department of Public Health and Nursing, Norwegian University of Science and Technology, Postbox 8905, 7491 Trondheim, Norway; 2 Center for Health Promotion Research, Norwegian University of Science and Technology, Trondheim, Norway

**Keywords:** Mental health literacy, Adolescence, Measurement, Health promotion

## Abstract

**Background:**

Mental health literacy (MHL), or the knowledge and abilities necessary to benefit mental health, is a significant determinant of mental health and has the potential to benefit both individual and public mental health. MHL and its measures have traditionally focused on knowledge and beliefs about mental -ill-health rather than on mental health*.* No measures of MHL addressing knowledge of good or positive mental health have been identified. *Aim:* This study aimed to develop and validate an instrument measuring adolescents’ knowledge of how to obtain and maintain good mental health and to evaluate the psychometric properties of the instrument. More specifically, the factor structure, internal and construct validity, and test-retest reliability were assessed.

**Methods:**

The participants were Norwegian upper secondary school students aged 15–21 years. The development and validation of the instrument entailed three phases: 1) item generation based on the basic psychological needs theory (BPNT), focus group interviews, and a narrative literature review, 2) a pilot study (*n* = 479), and 3) test-retest (*n* = 149), known-groups validity (*n* = 44), and scale construction, item reduction through principal component analysis (PCA), and confirmatory factor analysis (CFA) for factor structure and psychometric properties assessment (*n* = 1888).

**Results:**

Thirty-two items were initially generated, and 15 were selected for the pilot study. PCA identified cross-loadings, and a one-factor solution was examined. After removing five problematic items, CFA yielded a satisfactory fit for a 10-item one-factor model, referred to as the mental health-promoting knowledge (MHPK-10) measure. The test-retest evaluation supported the stability of the measure. McDonald’s omega was 0.84, and known-groups validity test indicated good construct validity.

**Conclusion:**

A valid and reliable one-dimensional instrument measuring knowledge of factors promoting good mental health among adolescents was developed. The instrument has the potential to complement current measures of MHL and may be useful when planning mental health promotion activities and evaluating public mental health education initiatives in adolescents.

**Electronic supplementary material:**

The online version of this article (10.1186/s12889-017-4733-6) contains supplementary material, which is available to authorized users.

## Background

Mental health in the Norwegian adolescent population has received considerable attention in recent years and has emerged as a public health concern that needs to be addressed [1]. Adolescence is considered an important transitional period in life during which individuals are particularly sensitive to contextual and surrounding influences; this unique state leads to challenges but also opportunities for improving health [2].

Health literacy (HL) concerns adolescents’ capacity to make sound health decisions in the context of their everyday lives [3] and is considered critical for effective participation in health promotion [4]. HL is a multifaceted, complex and evolving concept, and Sørensen et al. [5] has developed a definition and conceptual model relevant for the further work of conceptualization and measure development for *mental* health literacy (MHL).

MHL is a component of HL and is also an evolving concept. MHL is considered a significant determinant of mental health and has the potential to benefit both individual and public mental health [6, 7]. MHL has been conceptualized in different ways since the term was first coined by Jorm and colleagues in 1997 [6, 8–10]. Traditionally, MHL and its measures have focused on knowledge and beliefs about mental -ill-health rather than on mental *health* [11]. However, in past years, MHL has evolved from a focus on mental -ill-health and risk factors to providing an asset for health that can be strengthened through educational initiatives [7]. Today, MHL broadly refers to the knowledge and abilities necessary to benefit mental health [9]. A recent definition of MHL outlines four key components:

*“(1) Understanding how to obtain and maintain good mental health; (2) understanding mental disorders and their treatments; (3) decreasing stigma related to mental disorders; (4) enhancing help-seeking efficacy (knowing when, where, and how to obtain good mental health care and developing competencies needed for self-care)”*

(Kutcher et al. [12]).

This conceptualization advances previous perceptions of MHL as merely knowledge of mental disorders and is in line with the WHO’s definition of mental health, which states that mental health is more than the absence of mental disorders and includes wellbeing, optimal functioning and coping [13].

Several scales have been developed to capture the broad scope of MHL [8, 14–16]. However, the existing measures mainly address knowledge of the three latter components, namely mental disorders, stigma and help-seeking behaviors; no studies address knowledge of good or positive mental health [6]. Thus, a gap remains between the recent conceptualization of MHL [12] and available MHL measures. An instrument that rigorously measures the positive aspect of MHL can help determine a population’s or individual’s level of knowledge of factors promoting mental health. Furthermore, it could be used to evaluate interventions and educational initiatives to increase our understanding of the positive aspects of MHL and its associations with good mental health in adolescents.

A major challenge to developing a measure that assesses knowledge of factors promoting mental health is that individuals’ conceptions of what is needed to obtain and maintain mental health are highly individualized. However, there are known commonalities of the factors essential for obtaining and maintaining mental health. In this study, the basic psychological needs theory (BPNT) [17] was utilized to ground the measure in dimensions that are theoretically known to be important to good mental health and has been identified as an applicable conceptual framework for studying health-related behavior [18]. According to the BPNT, good mental health can be predicted by three dimensions: competence, autonomy and relatedness [17]. Competence refers to experiencing mastery and effectiveness in managing one’s environment. Autonomy refers to a sense of free will or acting out of one’s own interests and values. Finally, relatedness addresses the desire to interact with, feel a connection to, and care for other people [19].

The aim of the current study was to describe the development of an instrument measuring adolescents’ understanding of how to obtain and maintain good mental health (in this study referred to as mental health-promoting knowledge or MHPK) to represent MHL and to evaluate its psychometric properties. More specifically, the aims were to evaluate the factor structure, internal and construct validity, and test-retest reliability of the instrument.

## Methods

**Fig. 1:**
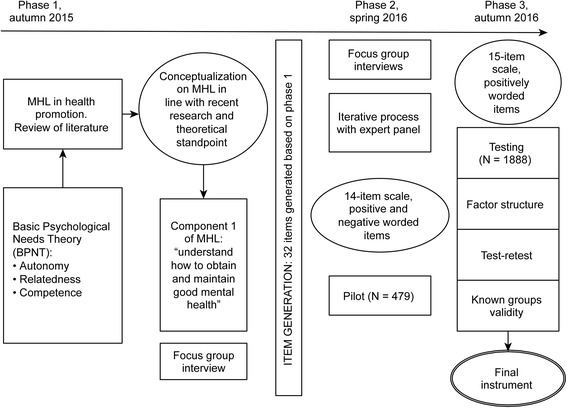
Description of timeline and phases of development of the instrument measuring mental health-promoting knowledge (MHPK)

The instrument was referred to as the MHPK and was developed in a three-step process (Fig. 1).

### Phase 1: item generation

Items were generated through a deductive approach using BPNT as the theoretical foundation [17, 20]. BPNT was applied during the development of the instrument measuring the positive component of MHL, i.e., component one in the recent definition, to identify the factors that actively promote mental health. In addition, a review of the literature on MHL and the seven rights of mental health (identity, meaning, mastering, belonging, safety, participation and sense of community) [21] were utilized to conceptualize knowledge of good mental health when generating relevant items. Thirty-two items were initially generated. Item generation was grounded in the three dimensions of BPNT and based on the narrative literature review and focus group discussions with adolescents. With the 32 items, all three dimensions of the BPNT were covered by a minimum of 7 items each, and recurrence in item proposals was observed. Hence, the decision was made within the research group to begin working with the pool of 32 items.

#### Focus group interviews

To explore and include adolescents’ perceptions of good mental health, five focus group interviews were conducted [22]. Adolescents aged 15–21 years from four upper secondary schools in an urban area in mid-Norway participated in the discussions in phase 1 (Fig. 1). Participants were recruited through the schools’ student councils by self-selection. There were 6–10 participants in each group, and both genders were represented, with a preponderance of girls. Semi-structured interview guides were developed in advance and used during the discussions. Mental health, factors important for good mental health, and items from the MHPK scale were discussed with the adolescents during an approximately one-hour session. The focus group discussions were transcribed, and adolescents’ collective perceptions of items and factors important for good mental health were extracted and used in scale development. No further analysis of the focus group discussions was performed for the purpose of scale development.

#### Expert panel

For content validation, an expert panel was invited to participate in the study (*n* = 10). Three public health nurses and six researchers within the field of health promotion (*N* = 9) provided iterative feedback during item development. Invitations to participate in the expert panel were extended to authors’ associates with appropriate academic qualifications and professional expertise in the field of mental health and school health services. The expert panel was asked to categorize items within the dimensions of competence, relatedness and autonomy. The items were included only if they were categorized in the same dimension and considered relevant by >7 members of the expert panel. This categorization eventually resulted in the inclusion of 15 items in the pilot study.

### Phase 2: pilot testing

A questionnaire including the 15-item scale was piloted at one upper secondary school in phase 2 (Fig. 1). Following informed consent from the principal to pilot the questionnaire at the designated school, each teacher chose whether they wanted to administer the survey to their class. The questionnaire was then administered by teachers over a two-week period; the teachers chose the session for administering the questionnaire at their convenience. The questionnaire was then given to 490 of 1075 students (46%); *n* = 479 (98%) responded. Pilot data were explored using Stata [23], and initial principal component analyses (PCA) were performed. Based on the focus group interviews, the expert panel comments and the pilot study results, two reversed items were deleted (e.g., in PCA, negative items generated a separate factor), one item was reworded, and two new items were added. In addition, “don’t know” was added as a response option. A 15-item scale was constructed and evaluated in phase 3 of the present study.

### Phase 3: participants

#### Sample 1

Over a three-week period in August 2016, a cross-sectional classroom survey was conducted at five upper secondary schools in an urban area in mid-Norway. Three of these five schools also participated in Phase 1, and one school also participated in the pilot study.

**Fig. 2:**
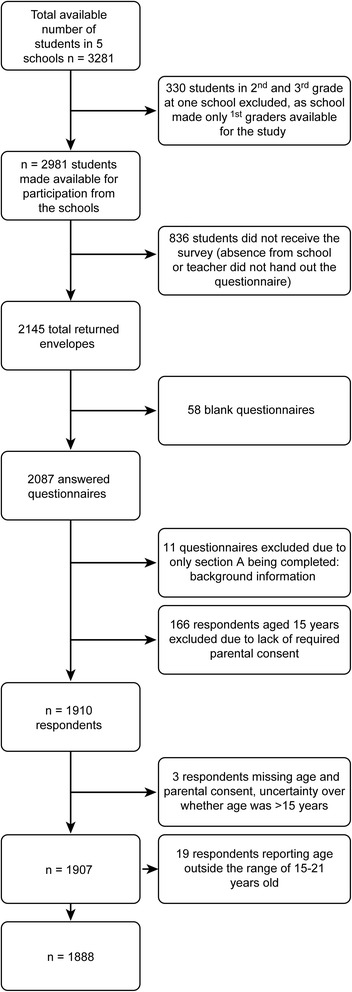
Flowchart of participants from sample 1: August 2016 student population

The questionnaire was administered to 2145 of 3281 students (65.4%), and *n* = 2087 (97.3%) responded with usable information (Fig. 2). The ages of the respondents ranged from 15 to 21 years. Seventy-four (3.9%) respondents did not report their age, and missing age values were replaced with the mean age of the sample (M = 17.02, SD = 1.04). The adolescent sample consisted of 51% girls and 49% boys; 62% of the students were from the “general studies” stream, whereas 38% of students were from “vocational studies” stream. When asked about their parents’ education, 38% of the adolescents responded “do not know”; 4.8% reported that their parents had received primary school education; 19%, upper secondary school; 21.7%, less than 4 years of university education; and 25.8%, more than 4 years of university education.

#### Procedure

Principals gave informed consent for data collection at the designated schools. Information regarding participation was provided by the research team through the schools’ teachers, and parents and students received written information letters. Furthermore, a short informational video was available to all participants on the schools’ e-learning platform (i.e., “it’s learning”). Teachers were responsible for allocating time and administering the survey, including reading aloud an information letter provided by the research group that stated that participation was voluntary and anonymous. Students aged 16 years or older gave consent for participation by completing the questionnaire, whereas written parental consent for students aged 15 was obtained. The study was approved by the Regional Committee for Medical and Health Research Ethics (REK midt 2014/1996).

#### Sample 2: known-groups validity

To further validate the instrument, third-year nursing students from the Norwegian University of Science and Technology (NTNU) were asked to respond to the MHPK instrument to complete a known-groups validity test. Third-year nursing students are expected to have higher levels of knowledge of the factors promoting mental health than adolescents aged 15–21 years, considering their educational background in mental health and health promotion. The instrument was distributed at the end of a regular lecture, and students who wanted to answer (*n* = 44) returned the completed MHPK before they left class, thereby forming a discretionary sample. The Norwegian Social Science Service (NSD) approved the inclusion of nursing students to test known-groups validity.

#### Measure

The MHPK scale was included as part of a questionnaire covering mental health and school health services. The MHPK measure consisted of 15 items representing statements of factors important to positive mental health; respondents were asked to rate each item on a six-point scale ranging from 1, “completely wrong”, to 5, “completely correct”, in addition to 0, “don’t know”.

### Statistics

STATA version 14.2 (StataCorp. 2015, Stata Statistical Software: Release 14, College Station, TX: StataCorp LP [23]) and Microsoft Excel (2011, version 14.7.1) were used for statistical analyses.

The 15 items were initially analyzed using principal component analysis (PCA) orthogonal rotation by default to explore the factor structure and identify split loadings to reduce items; 0.32 was set as the minimum factor loading, and loadings >0.55 were considered good [24]. Data were examined for normality, frequency and patterns of missing data. Testing for normality revealed significant kurtosis and skewed data (*p*-value <0.001). The data were determined to have a non-normal distribution, and thus Satorra-Bentler (robust to non-normality) was used as an estimation method in the confirmatory factor analysis (CFA) [25, 26]. CFA was performed to evaluate the model fit of the factor structure based on the PCA and to identify problematic items by inspecting modification indices (MI). Two different models were estimated to find the best fit: a 10-item version with a one- or three-factor solution. The fit indices assessed with cut-off values included the following: Chi-square test (χ^2^) to evaluate the global model fit; the Comparative Fit Index (CFI) and Tucker-Lewis Index (TLI), with values >0.90 considered adequate (preferably >0.95) [26]; Root Mean Square Error of Approximation (RMSEA), with cut-off values of <0.8 (preferably <0.5) [26]; and Standardized Root Mean square Residual (SRMR), where values <0.10 were considered acceptable [27]. Inter-item correlations and correlations between factors were evaluated using Pearson’s r. McDonald’s omega was calculated to evaluate the internal consistency of the measure [28].

### Missing values

All items were examined for missing values. In total, 94.6% of adolescents (*n* = 1786) responded to all items, 3.4% were missing one or two items, and the remaining 2% were missing 3–14 items. Items were missing at random and were evenly distributed across the scale, ranging from 0.9% to 2.5% on each item. Cases were deleted listwise.

### Test-retest reliability

Three weeks after the initial data collection, the instrument was administered to a discretionary sample subgroup (*n* = 219) of the original sample to evaluate the test-retest reliability of the instrument using Pearson’s correlation coefficient, r. A test-retest correlation coefficient above 0.70 was considered acceptable.

### Known-groups validity

To test the construct validity of the instrument, a known-groups validity test was performed; specifically, a two-tailed independent samples t-test was conducted to evaluate the mean group differences between the adolescent/student sample and the sample of nursing students. To evaluate the strength of the differences in mean scores between the adolescents and nursing students, the effect sizes were interpreted using Cohen’s d [29].

## Results

### Principal component analysis

Fifteen items were included in the PCA. Parallel analysis, eigenvalues and scree plots were used to determine how many factors should be retained after PCA. Two factors had eigenvalues above 1 (4.8 and 1.2), and the scree plot leveled off immediately after the two factors [24, 27]. Factor 1 explained 48% of the variance, while factor 2 accounted for 12% of the variance; the other factors explained very little of the variance in the 15 variables. PCA revealed five problematic items (items 1, 3, 4, 9 and 10), referring to a split loading >0.32 on two factors. Items were removed after evaluation of the split loadings and careful consideration of the item content (Table 1). Removing the five initially problematic items resulted in a 10-item one-factor solution in the PCA that explained 41% of the variance; the other factors explained <10% of the total variance. The 10-item version was referred to as the MHPK-10 (a copy of the MHPK-10 can be found as Additional file 1). The results of bivariate correlations showed significant inter-item correlations of the 10 items ranging from *r* = 0.29 to 0.52.

**Table 1:** Items, descriptive statistics and factor loadings in PCA

Items	Mean	Split factor loadings 15-item version: *n* = 1786		Factor loadings 10-item version: *n* = 1813	Intended theoretical dimension
		Factor1	Factor2		
1. Having at least a good friend		0.50	0.56		Relatedness
2. Handling stressful situations in a good manner	4.20			0.62	Competence
3. Having influence on your own day		0.60	0.37		Autonomy
4. Acting out of your own wishes		0.59	0.44		Autonomy
5. Believing in yourself	4.62			0.70	Competence
6. Having good sleep routines	4.18			0.63	Competence
7. Making decisions based on own will	4.39			0.59	Autonomy
8. Setting limits for your own actions	4.30			0.66	Autonomy
9. Being a good friend		0.67	0.36		Relatedness
10. Feeling safe at home		0.70	0.42		Relatedness
11. Feeling that you belong in a community	4.58			0.66	Relatedness
12. Mastering your own negative thoughts	4.20			0.72	Competence
13. Setting limits for what is OK for me	4.41			0.72	Autonomy
14. Feeling valuable regardless of your own accomplishments	4.20			0.74	Relatedness
15. Experiencing school mastery	4.10			0.68	Competence
Explained variance per factor		48%	12%	41%	

BPNT was used for item development and included three dimensions: competence, relatedness and autonomy

Frequency N per item range was 1840–1871

Cases were deleted listwise

### Confirmatory factor analysis of the final 10-item version

Following the PCA, the 10-item version was assessed in CFA to evaluate the factor structure. Two models were tested. The one-factor model was based on the PCA and referred to the concept of MHPK as one component of MHL. The three-factor model was based on the theoretical foundation of BPNT and its three dimensions for item development.

**Fig. 3:**
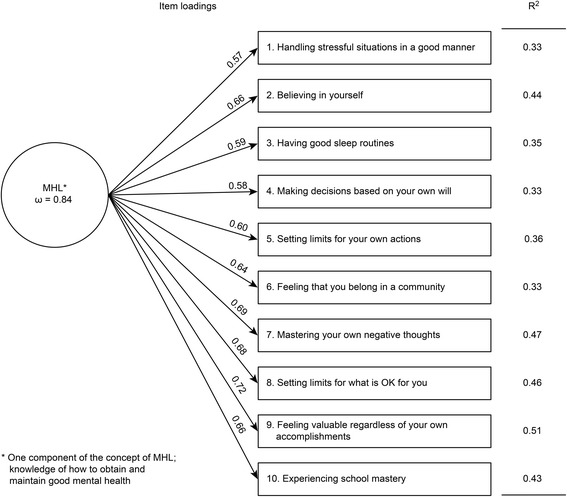
The mental health promoting-knowledge instrument (MHPK-10). *Legend: McDonald’s omega, item loadings (standardized), and explained variance (R* ^2^ *) for the one-factor model representing one component of MHL*. *n* = 1813. Reliability coefficient = 0.874

The model fit indices presented in Table 2 reveal a slightly better fit for the three-factor model than for the one-factor model; however, both models showed a decent fit to the data. The correlations between factors in the three-factor model were strong (0.82 to 0.97), and the one-factor model representing the positive component of MHL was theoretically preferred. A final 10-item one-factor model was therefore estimated and exhibited an adequate model fit (Fig. 3).

**Table 2:** Fit indices for CFA models. One- and three-factor solutions for the 10-item version of the MHPK

MHPK 10-item version							
Model	χ^2^	df	χ^2^ /df	CFI	RMSEA	SRMR	TLI
Single-factor	169.41^a^	35	4.84	0.946	0.046	0.035	0.930
Three-factor	114.25^a^	32	3.57	0.967	0.038	0.027	0.953

Note: All *p* values are statistically significant (*p* < 0.001)^a^; *n* = 1813

McDonald’s omega (*ω* = 0.84) of the one-factor model was high, indicating support for the internal consistency of the one-factor solution based on the measure’s internal structure [28]. The final 10-item one-factor model showed good to excellent factor loadings (0.57–0.72) and a reasonably good fit to the data (Fig. 3). Inspection of MIs indicated that item 8 covaried with several other items, revealing a covariance MI of 75.9 with item 7. After removing item 8, all MIs were ≤20. However, as the preset criteria for model fit were met, no modifications based on MIs were made [30].

### Test-retest

The test-retest coefficient for the 10-item version was *r* = 0.74, indicating acceptable reliability of the instrument according to the a priori established cut-off value of 0.70.

### Known-groups validity

The results of the independent samples t-tests showed that nursing students (M = 4.69, SD = 0.33) scored significantly higher on the scale than adolescents (M = 4.51, SD = 0.54), *t* = 2.2012, *p* = 0.0278. The difference in mean scores between adolescents and nursing students was 0.18 and of medium strength (Cohen’s d = 0.40). The results support the instrument’s construct validity by showing that third-year nursing students scored significantly higher than adolescents on the measure of factors promoting good mental health, as expected.

### Descriptive statistics for the MHPK-10

The adolescent population was used to generate descriptive statistics for the MHPK-10. The mean score was 4.51 (SD = 0.54 Minimum = 0, Maximum = 5, 95% CI = 4.29–4.53). The results of the MHPK-10 showed that 19.33% of the student population had an insufficient level of knowledge of factors promoting good mental health (a mean score < 4 was used as a preliminary cut-off for an insufficient level of knowledge since values 4 and 5 identifies the correct answer to each statement; however, this cut-off must be further evaluated). In terms of analyses using the MHPK-10, we suggest using mean scores and allowing two missing items per respondent.

## Discussion

In this study, we successfully developed a valid and reliable instrument that measures adolescents’ knowledge of factors promoting good mental health. In promoting mental health, there has been a shift from a problem-focused approach emphasizing the prevention of psychological distress and viewing mentally negative conditions as illnesses toward a greater focus on resources contributing to positive development and wellbeing [31]. Consequently, the positive conditions and factors involved in mental health promotion need to be assessed with relevant and psychometrically sound measures, such as measures of MHL. MHL has not been consistently conceptualized in the literature, and thus no gold standard for measuring the concept exists [14]. However, in the field of HL, complex conceptual models have been developed [5]. These models are useful to place MHL in a wider context, but it is important to emphasize that we do need a domain-specific approach for MHL to draw attention to a neglected area [10]. Mental health is an integral part of health [13], thus MHL may be merged with HL in the future, but for now, there is a need for a domain-specific approach for mental health to specifically meet the need for tailored measures in the evaluation of MHL interventions [10] and assessment of MHL levels in populations. The MHPK-10 measure is a contribution to the field of MHL and is based on the most recent conceptualization of MHL [14, 16]. When discussing the relationship between knowledge and health behavior in the final paragraph of this discussion section, MHPK will be discussed in the context of Sørensen et al.’s conceptual model of HL.

### Development of the instrument

The approach used to develop the instrument followed scientifically accepted principles [20]. The major challenge was developing items that detected knowledge within the scope of MHL in health promotion without being overly intuitive. Intuitive items might have led to artificially high mean scores, subsequently missing adolescents with low knowledge of factors promoting good mental health. The results were skewed, which may be because the population had high levels of knowledge or because the scale was too intuitive. It is challenging to cover all aspects of MHPK because individuals’ conceptions of what is needed to strengthen mental health vary. However, the items were based on a solid theoretical foundation, adolescents’ opinions and acknowledged expertise to ensure a solid grounding for the instrument. Combined with the empirically determined seven mental health rights [21], substantial groundwork was established to ensure that the instrument had a theoretical and empirical foundation to measure factors promoting good mental health. Thus, the MHPK-10 is considered a solid starting point for further development and validation of a measure assessing one component of MHL: knowledge of how to obtain and maintain good mental health.

### Factor structure

The PCA yielded support for a one-factor solution of the 10-item version of the MHPK reflecting component one (understanding how to obtain and maintain good mental health [7]) of the four components included in the definition of MHL. A three-factor structure was also evaluated considering the three dimensions of BPNT utilized to generate the items. The fit indices showed a slightly better fit for the three-factor model than for the one-factor model. However, the covariance between the three factors was high, and a one-factor solution corresponded better with the instrument as a measure of one component of MHL. Furthermore, the dimension relatedness in the three-factor solution was problematic and consisted of only two items. Thus, the instrument was constructed as a one-factor model with good to excellent factor loadings and a good overall McDonald’s omega value corresponding to the intended component of MHL.

### Evaluation of validity and reliability

According to the results, the reliability of the instrument was acceptable. The MHPK-10 demonstrated good internal validity and test-retest reliability. The test-retest analyses were used to assess the consistency and sensitivity of the measure over time. The known-groups validity test showed that the instrument was able to differentiate between those who were expected to have greater knowledge of factors promoting good mental health based on their university education and those who were expected to have less knowledge, namely, upper secondary school students. However, when examining the actual variance of the results, the differences in mean scores were small. Both groups had acceptable levels of knowledge. This finding can be interpreted as variations within the accepted level of knowledge that could be of clinical and practical relevance in mental health promotion. At this point, the instrument should not be used solely to detect whether a population has sufficient knowledge of factors promoting health but rather to identify the fraction of the population lacking this knowledge and areas in need of public mental health education.

### Strengths and limitations

One strength of the current study is the large sample size and high response rate. A sound psychometric evaluation was performed to assess the MHPK-10 measure; the findings contribute to the field by enabling future use and call for further development and validation of the instrument. However, the results should be interpreted with some caution. The focus groups may have been subject to self-selection bias. The instrument may therefore be overly influenced by females’ opinions on mental health, given the preponderance of girls in the focus groups. However, males were represented and contributed opinions and experiences in all focus groups and in the expert panel. For the survey, teachers served as administrators of the questionnaire and thus may have influenced which classes had the opportunity to participate in the study by serving as gatekeepers for student participation.

Finally, the MHPK-10 score distributions showed little variance in the mean scores and hence demonstrate a possible ceiling effect, indicating that the measure in its current form does not sufficiently discriminate among adolescents with high MHPK-10 levels. This ceiling effect may cause difficulties in establishing the discriminant validity of the scale.

### Implications and future research

Future research should further refine the MHPK-10 to be less intuitive, thus yielding more variance in the item responses and reduce ceiling effects. Further testing of the scale is needed to evaluate the cut-off values for sufficient knowledge of factors important for good mental health. Further validation of the instrument across samples and age groups is also needed. The implications for adolescent mental health may include that the MHPK-10 identifies mental health promotion areas with low levels of knowledge in populations. Public health practitioners could subsequently target their mental health education toward the aspects with an identified need in particular populations. The MHPK-10 also has the potential to be used to evaluate mental health-promoting education initiatives aimed at increasing knowledge of factors promoting mental health to improve and better tailor these initiatives. As MHL is considered an outcome of mental health promotion actions, every item in the MHPK-10 is considered applicable and translatable into public health practice; i.e., mental health education can be developed to improve knowledge of any of the items [32]. The MHPK-10 identified approximately 20% of the student population as having inadequate knowledge of factors promoting good mental health. This finding corresponds well with established numbers on adolescents’ mental health status; specifically, 15–20% of Norwegian adolescents report mental health issues [1]. An important next step is to study the relationship between the MHPK-10 and mental health, in particular, how levels of knowledge of factors promoting mental health are related to self-reported mental health and mental health-promoting behavior.

### MHL and health-promoting behavior

It is important to consider the difference between adolescents’ knowledge of the factors that promote mental health and their possession of the skills to apply that knowledge. With respect to the process of applying the knowledge detected by the MHPK-10, Sørensen et al.’s HL model is a relevant model. Sørensen et al.’s model introduces competencies, knowledge and motivation of how to access, understand, appraise and apply health-related information as central in HL [4, 5]. According to the model, health-related knowledge empowers people to participate in health-promoting activities in communities [5], e.g., in a school setting. The MHPK-10 instrument measures knowledge of factors important to obtain and maintain good mental health on an individual level for use in a public health perspective. Although knowledge of these factors or mental health literacy does not necessarily lead to mental health-promoting behavior, we argue that knowledge is as a necessary foundation for making purposeful health-promoting decisions, in line with Sørensen et al.’s conceptual model of HL [5]. HL is known to affect health behavior and consequently health outcomes [4], and we expect MHL to have similar impact. Although knowledge does not necessarily mean skills, again, we argue that knowledge is fundamental for building skills to apply knowledge and a necessary starting point for promoting mental health among adolescents.

## Conclusion

This study found support for a valid and reliable 10-item one-factor measure of adolescents’ knowledge of factors promoting good mental health. A rigorous evaluation of the scale’s psychometric properties was performed, and satisfactory internal consistency and construct validity were established. The MHPK-10 is a solid first step toward creating a sound measure of the positive aspects of MHL and has the potential to complement current measures of MHL for use in health promotion. The results are particularly applicable for guiding the development, targeting and evaluation of public mental health education initiatives, with the main goal of building a foundation for good mental health, wellbeing and productive future lives for adolescents. The MHPK-10 measure provides a novel contribution and requires further refinement and validation.

## Additional file

Additional file 1: MHPK-10 instrument. A copy of the 10-item MHPK instrument that measures adolescents’ knowledge of how to obtain and maintain good mental health. (PDF 105 kb)

## Abbreviations

BPNT: Basic psychological needs theory
MHL: Mental health literacy
MHPK: Mental health-promoting knowledge
