# Supplementary material for: Positive mental health literacy: development and validation of a measure among Norwegian adolescents
Source: BMC Public Health. 2017 Sep 18;17:717. doi: 10.1186/s12889-017-4733-6 (PMC5604188; doi:10.1186/s12889-017-4733-6)
Supplement: Additional file 1: — MHPK-10 instrument. A copy of the 10-item MHPK instrument that measures adolescents’ knowledge of how to obtain and maintain good mental health. (PDF 105 kb) [file 12889_2017_4733_MOESM1_ESM.pdf]

WHAT IS IMPORTANT FOR GOOD MENTAL HEALTH?

Here are 10 statements about things that can be important for good mental health. On the scale from 1 to 5, how correct is each statement?

	Completely wrong 1	*Slightly wrong* 2	*Neither /nor* 3	*Slightly correct* 4	*Completely correct* 5	*Don't know* ⇩
1. Handling stressful situations in a good manner	☐	☐	☐	☐	☐	☐
2. Believing in yourself	☐	☐	☐	☐	☐	☐
3. Having good sleep routines	☐	☐	☐	☐	☐	☐
4. Making decisions based on your own will	☐	☐	☐	☐	☐	☐
5. Setting limits for your own actions	☐	☐	☐	☐	☐	☐
6. Feeling that you belong in a community	☐	☐	☐	☐	☐	☐
7. Mastering your own negative thoughts	☐	☐	☐	☐	☐	☐
8. Setting limits for what is OK for you	☐	☐	☐	☐	☐	☐
9. Feeling valuable regardless of your own accomplishments	☐	☐	☐	☐	☐	☐
10. Experiencing school mastery	☐	☐	☐	☐	☐	☐
